# Predicting fluid responsiveness in non-intubated COVID-19 patients: two methods are better than one

**DOI:** 10.1186/s13613-021-00826-4

**Published:** 2021-02-15

**Authors:** Morgan Caplan, Michael Howsam, Raphael Favory, Sebastien Preau

**Affiliations:** 1 Division of Intensive Care, University of Lille, CHU Lille, 59000 Lille, France; 2 Inserm, Institut Pasteur de Lille, U1167, University of Lille, 59000 Lille, France; 3 Division of Intensive Care, Inserm, Institut Pasteur de Lille, U1167, University of Lille, CHU Lille, 59000 Lille, France

To the Editor,

We thank Dr. Michard for his constructive commentary on our recent publication, “Measurement site of inferior vena cava (IVC) diameter affects the accuracy with which fluid responsiveness can be predicted in spontaneously breathing patients” [1, 2]. Dr. Michard suggests that measuring the collapsibility index of the IVC (cIVC) to predict fluid responsiveness (FR) may be difficult in non-intubated patients with acute respiratory failure (ARF), and appears to favor the passive leg raising (PLR) maneuver to assess FR, in particular for obese patients [1].

We agree with him that there is a need for accurate predictions of FR in ARF patients, since fluid overload could be particularly harmful in this population. Nevertheless, we do not agree with his implication that cIVC cannot be used in ARF patients [1]. In the recent COVID-ICU cohort, 54% of 4244 adults admitted to ICU for COVID-19 were not intubated on ICU admission: standard oxygen therapy, high-flow oxygen, and non-invasive ventilation were applied to 29%, 19%, and 6% patients, respectively [3]. Among 81 patients included in our own study [2], 49 (61%) had sepsis of pulmonary origin and 60 (74%) had an ARF requiring oxygen administration: standard oxygen therapy and high-flow oxygen were administered to 50 (62%) and 10 (12%) patients, respectively (unpublished data). Moreover, with a median (interquartile) age of 64 (54; 73) years and a Simplified Acute Physiology Score (SAPSII) of 34 (24; 42), our patients were remarkably close to the “real life” conditions of patients from the COVID-ICU cohort.

We acknowledged in our article that the use of cIVC to predict FR is not valid in patients with non-invasive ventilation and clinical signs of active exhalation, and that PLR would be helpful in these situations. Nonetheless, we feel that moving ARF patients from a semi-recumbent position to a PLR position risks worsening their respiratory function. Since the individual tolerance of ARF patients to a PLR maneuver is difficult to predict, we suggest using cIVC, under the validated conditions described in our study, as the preferred predictive measure of preload-responsiveness and in order to avoid changing the patient’s position and potentially worsening ARF.

We also disagree with Dr. Michard’s assertion that cIVC, measured 4 cm caudal to the cavo-atrial junction, has limited value in patients making significant inspiratory efforts [1]. Bortolotti et al*.* [4] previously showed that the inspiratory effort was positively correlated with cIVC in responders but not in non-responders to volume expansion. Our study showed that a reduction in IVC diameter of > 33% during a non-standardized inspiration (cIVC-ns) predicted FR with low sensitivity but high specificity. In cases of low cIVC-ns values, therefore, FR is uncertain and use of a standardized significant inspiratory effort (cIVC-st) is to be recommended. In these cases, a cIVC-st of > 44% predicted FR with both high sensitivity and specificity [2]. Nevertheless, we acknowledged in our article that cIVC has its limitations in patients unable to make sufficient inspiratory efforts. Bortolotti et al*.* [4] reported that 5 (9%) patients were unable to reach an inspiratory pressure threshold of − 3 mmH_2_O, which inability was associated with an increased risk of false-negative results of cIVC-st, and underlines the importance of monitoring the buccal pressure in case of low values of cIVC-st.

Forty-one percent of patients in the COVID-ICU cohort were obese, and the median body mass index (BMI) of the overall population was 28 (25; 32) [3]. The median BMI of the patients included in our study was 25 (22; 29) [2], and among them 26 (32%) were overweight and 13 (16%) obese. The cIVC measurements were performed without systematic measurement of intra-abdominal pressure which is commonly higher among obese patients. While we acknowledged in our article that higher intra-abdominal pressure may influence IVC diameter and hence cIVC accuracy in predicting FR, the PLR test is similarly affected: for example, Beurton et al. [5] recently demonstrated that intra-abdominal hypertension is responsible for false negatives in the PLR test. Overall, we agree with Dr. Michard that obesity may affect the accuracy with which cIVC predicts FR [1], but obesity may also alter PLR-induced hemodynamic changes, and its influence on both cIVC’s and PLR’s accuracy in predicting FR in non-intubated patients has yet to be established.

In summary, both cIVC and PLR display significant limitations to predict FR in non-intubated COVID-19 patients. Moreover, while echography skills and ultrasound materials are required to assess both cIVC and PLR-induced changes in the velocity time integral of the aortic blood flow, specialized devices are required to track PLR-induced changes in stroke volume or surrogates. Notwithstanding these technical challenges, we are firmly of the opinion that both methods are viable approaches, and that their pragmatic and complementary use remains a useful strategy in assessing whether patients with ARF may benefit from rapid volume expansion in the presence of hypoperfusion (Fig. 1).

**Fig. 1:**
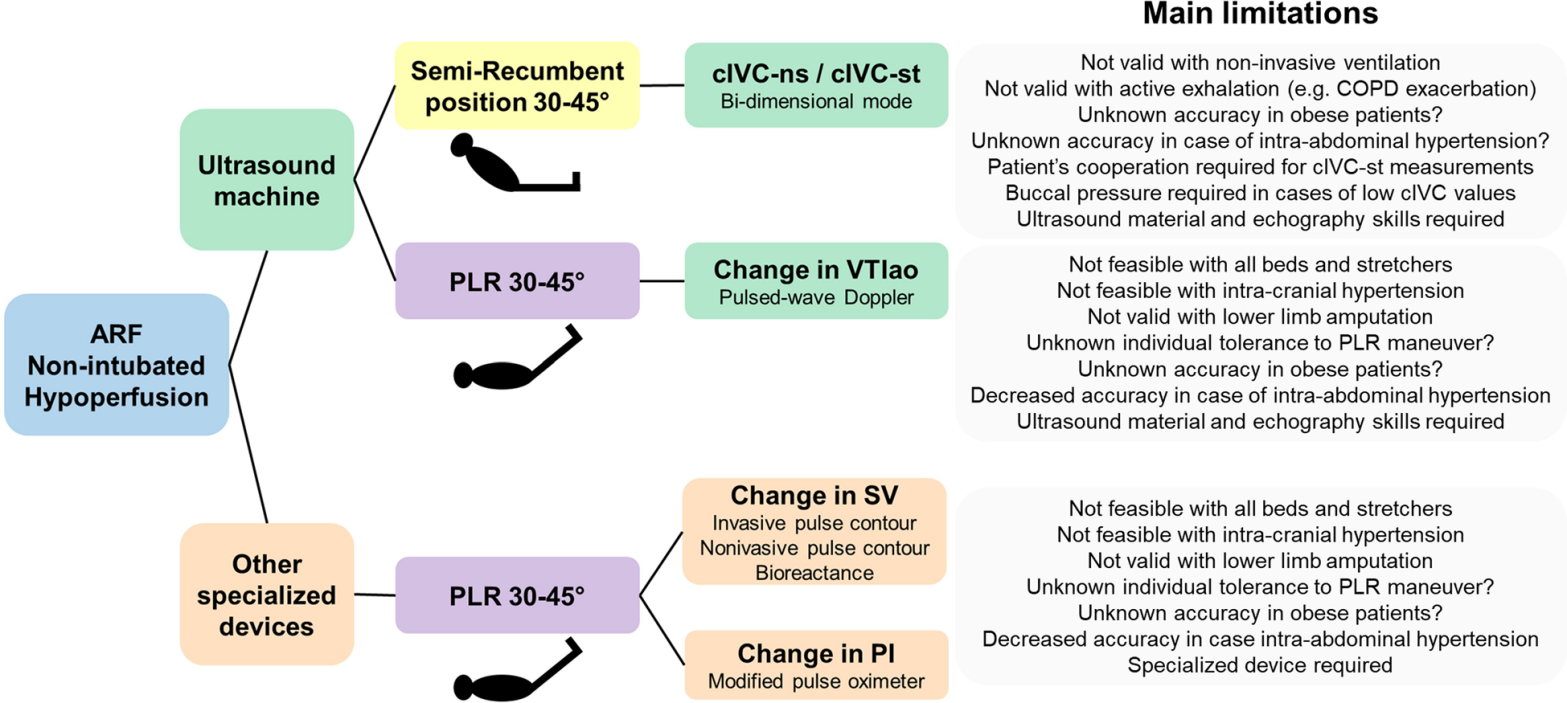
How to predict fluid responsiveness in non-intubated patients with hypoperfusion and acute respiratory failure (ARF). *cIVC* collapsibility index of the inferior vena cava with standardized (-st) and unstandardized (-ns) inspiratory manoeuver, *COPD* chronic obstructive pulmonary disease, *PI* perfusion index, *PLR* passive leg raising, *VTIao* velocity time integral of aortic blood flow

## Data Availability

The datasets used and/or analyzed during the current study are available from the corresponding author on reasonable request.
